# Supplementary material for: The CHD6 chromatin remodeler is an oxidative DNA damage response factor
Source: Nat Commun. 2019 Jan 16;10:241. doi: 10.1038/s41467-018-08111-y (PMC6335469; doi:10.1038/s41467-018-08111-y)
Supplement: Supplementary file 1 — Supplementary Information [file 41467_2018_8111_MOESM1_ESM.pdf]

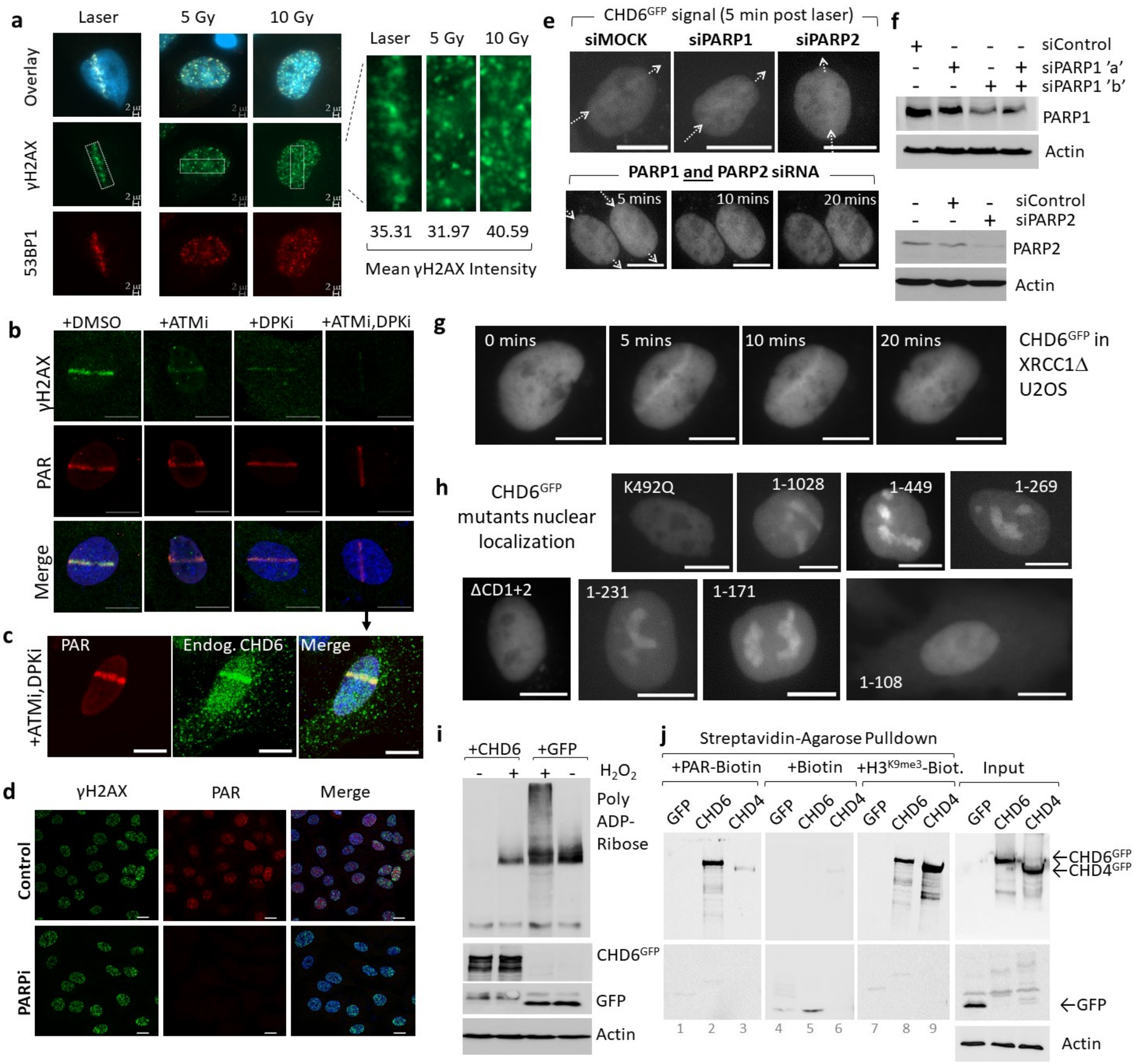

Supplementary Figure 1: CHD6 dynamically responds to oxidative DNA damage. A: A549 cells exposed to laser microirradiation or 5 or 10 Gy IR were immunostained for γH2AX (green) or 53BP1 (red) and DAPI (blue, in overlay). γH2AX intensity per area was quantified. Scale bars = 2 µM. B: A549 cells were treated with ATM and/or DNA-PKcs inhibitors for 1 h before being subjected to microirradiation, fixed 5 min later and immunostained for endogenous γH2AX (green) and PAR (red). Overlay includes DAPI (blue). Scale bars = 10 µM. C: Cells from (B) were also stained for endogenous CHD6 (green) and PAR (red). Scale bars = 10 µM. D: A549 were treated with 5 Gy IR ± PARPi and immunostained as in (B). Scale bars = 20 µM . E: Representative images of data in Fig 3c. Scale bars = 10 µM. F: A549 cells transfected with siRNA targeting indicated proteins (used in Fig 3) were immunoblotted to confirm protein knockdown. G: U2OS cells deleted for XRCC1 and transfected with $CHD6^{GFP}$ were subjected to laser microirradiation and live imaged over 20 min. Scale bars = 10 µM. H: Live imaging stills of GFP signal from A549 cells transfected with indicated GFP-tagged CHD6 constructs indicating nuclear localization. Some nucleolar accumulation is observed for truncation mutants. Only once the extreme N-terminus is perturbed (aa1-108) does CHD6 become

partially cytoplasmic. Scale bars = 10 μM. I: Extracts from HEK293 transfected with GFP or GFP-tagged CHD6 or CHD4 were incubated with strepatavidin-agarose beads loaded with biotin alone, biotin-PAR or biotin-H3^{K9me3}. Washed pull-downs were immunoblotted for indicated proteins. J: HEK293 cells were transfected with GFP or GFP-tagged CHD6 add, 16 h later, treated with 5 μM PARGi for 0.5h before the addition of 1 mM H_2O_2 for 1h. Whole cell extracts were immunoblotted for the indicated proteins. Representative blot shown, n=3.

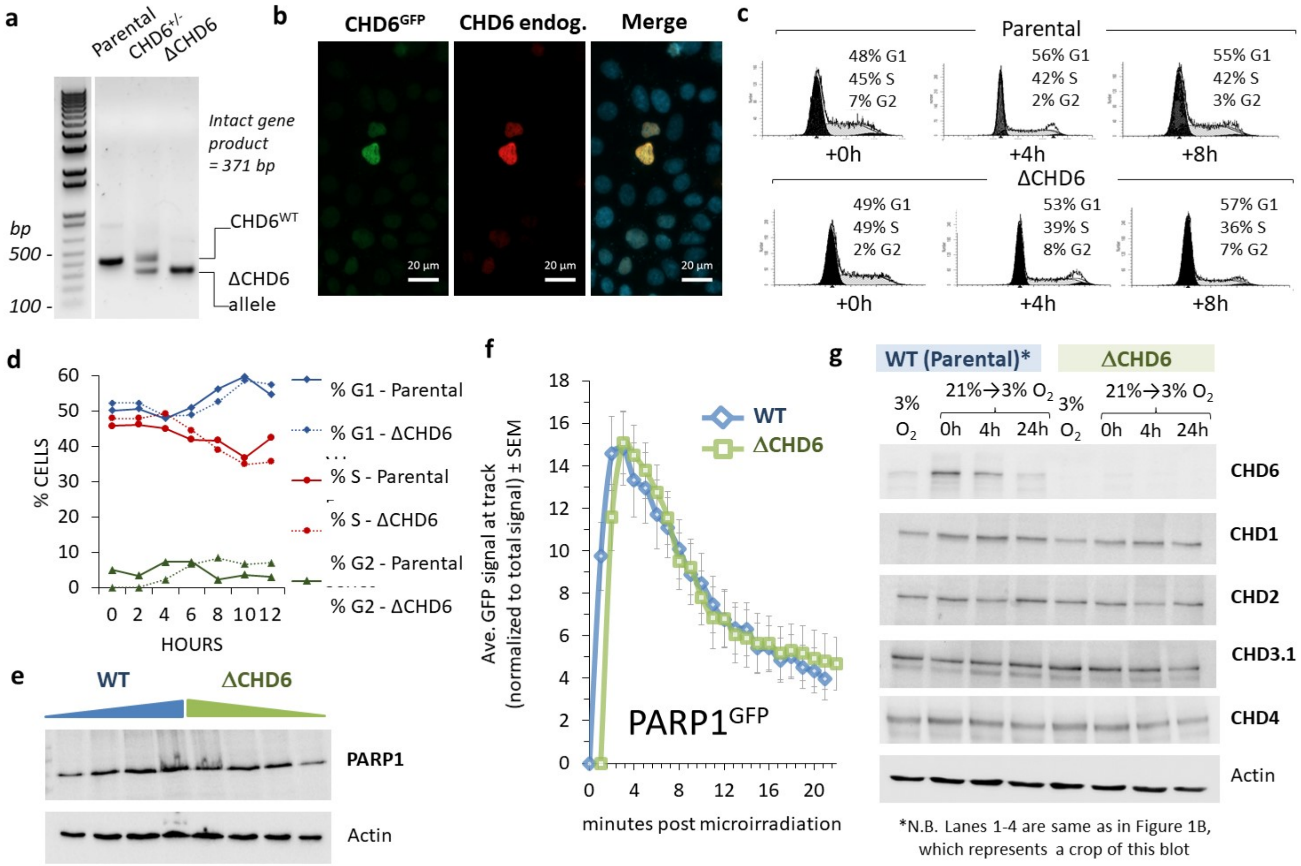

Supplementary Figure 2: Phenotypes of CHD6 ablated cells. A: Genomic DNA was isolated from wildtype 'parental' A549 cells and two single-cell cloned lines subjected to CRISPR-based gene editing to delete CHD6. The CHD6 cDNA was then analyzed by PCR to confirm presence of intended mutation, with the intact (wildtype) gene product predicted to be 371 bp and the product from the edited gene to be 100 nt smaller. B: ΔCHD6 A549 cells were transfected with CHD6^{GFP} (green) and immunostained for endogenous CHD6 (red). Scale bars = 20 μM. C-D: Fluorescence Activated Cell Sorting (FACS) based on DNA content was used to determine relative cell cycle phase distribution of the parental and ΔCHD6 cells over a 12h period. E: Increasing amounts (5, 10, 25, 50 μg) of whole cell extract from wildtype and ΔCHD6 A549 were immunoblotted for PARP1 and actin as indicated. F: GFP-tagged PARP1 was transfected into wildtype and ΔCHD6 A549 and subjected to laser microirradiation and live cell imaging. Data is plotted as in Fig 3. Error bars represent s.e.m, n=20. There is no significant difference between wildtype and CHD6-deleted cells. G: Wildtype and ΔCHD6 A549 were treated as in Fig 1A and immunoblotted for CHD1, CHD2, CHD3.1, CHD4 or CHD6 and actin. This blot represents the uncropped version of Fig 1b. In all cases, blue represents Parental WT A549s and green represents ΔCHD6 A549s.

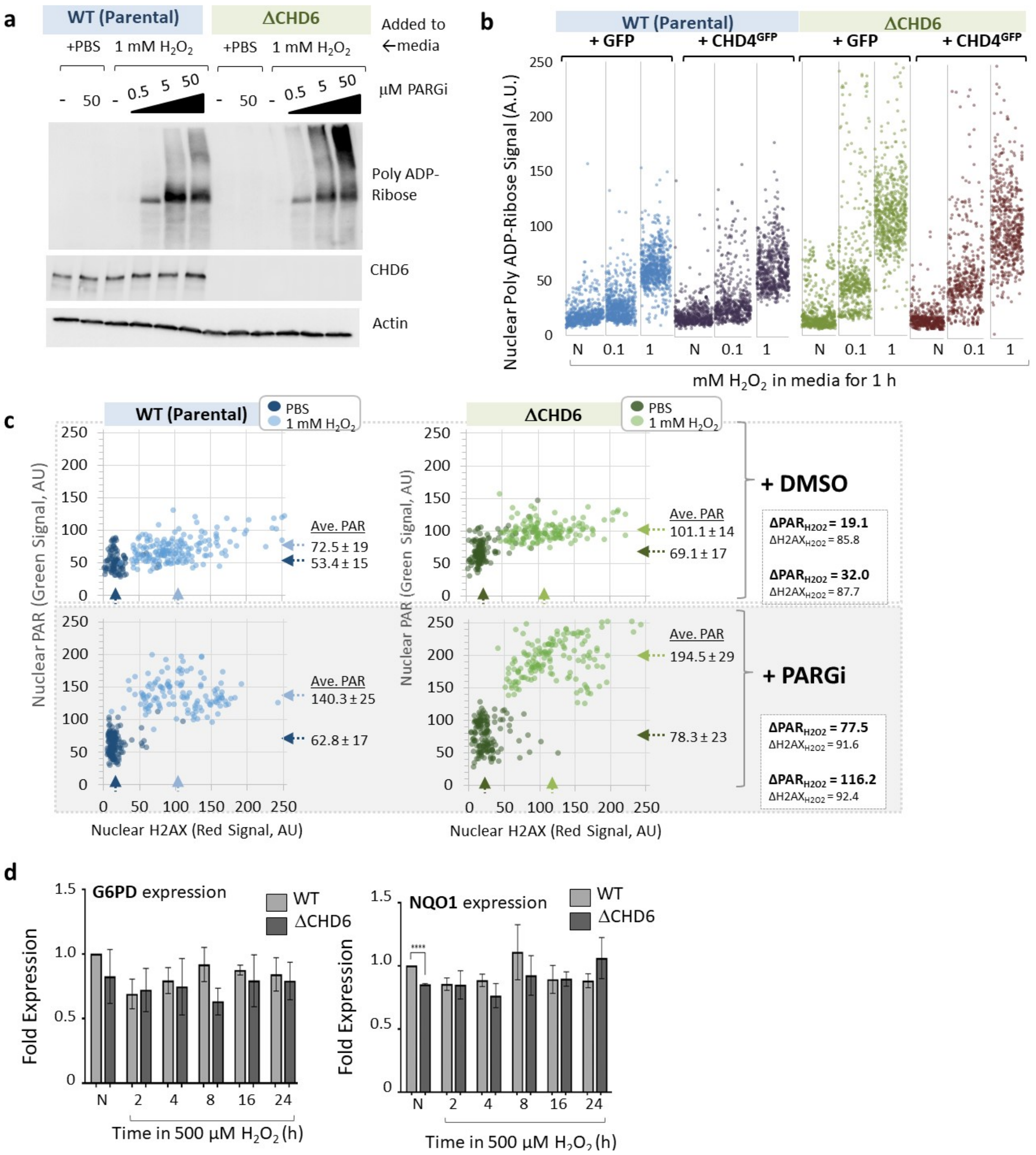

Supplementary Figure 3: Phenotypes of CHD6 ablated cells continued. A: WT and ΔCHD6 A549 cells were treated ± 1 mM H_2O_2 in media and indicated doses of PARGi for 1 h before being immunoblotted for Poly ADP-Ribose (PAR), CHD6 and Actin. B: WT (blue, purple dots) and ΔCHD6 (green, red dots) A549 cells were transfected with GFP or $CHD4^{GFP}$ before being treated with 5 µM PARGi and 0, 0.1, 1 mM H_2O_2 in media for 1 h and immunostained for Poly ADP-Ribose (PAR) and GFP. PAR signal in 300-400 (per experiment, n=3) GFP-positive cells was quantified by ImageJ. Purple represents WT A549s transfected with $CHD4^{GFP}$ and red represents ΔCHD6 A549s transfected with $CHD4^{GFP}$. C: WT and $A549^{\Delta CHD6}$ were treated with either DMSO or 5 µM PARGi ± 1 mM H_2O_2 for 1 h, immunostained for PAR and γH2AX and quantified by ImageJ. Nuclear PAR in >1,000 GFP-positive cells was quantified by ImageJ, n=3. D: WT (light grey) and ΔCHD6 (dark grey) A549 cells were exposed to 0 or 500 µM H_2O_2 in media for 1 day, before extraction and analysis by quantitative PCR to ascertain NQO1, G6PD and TBP mRNA expression. Error bars represent s.e.m, n=3. Students unpaired t-test, ****= $p<0.0001$. In all cases, blue represents Parental WT A549s and green represents ΔCHD6 A549s.

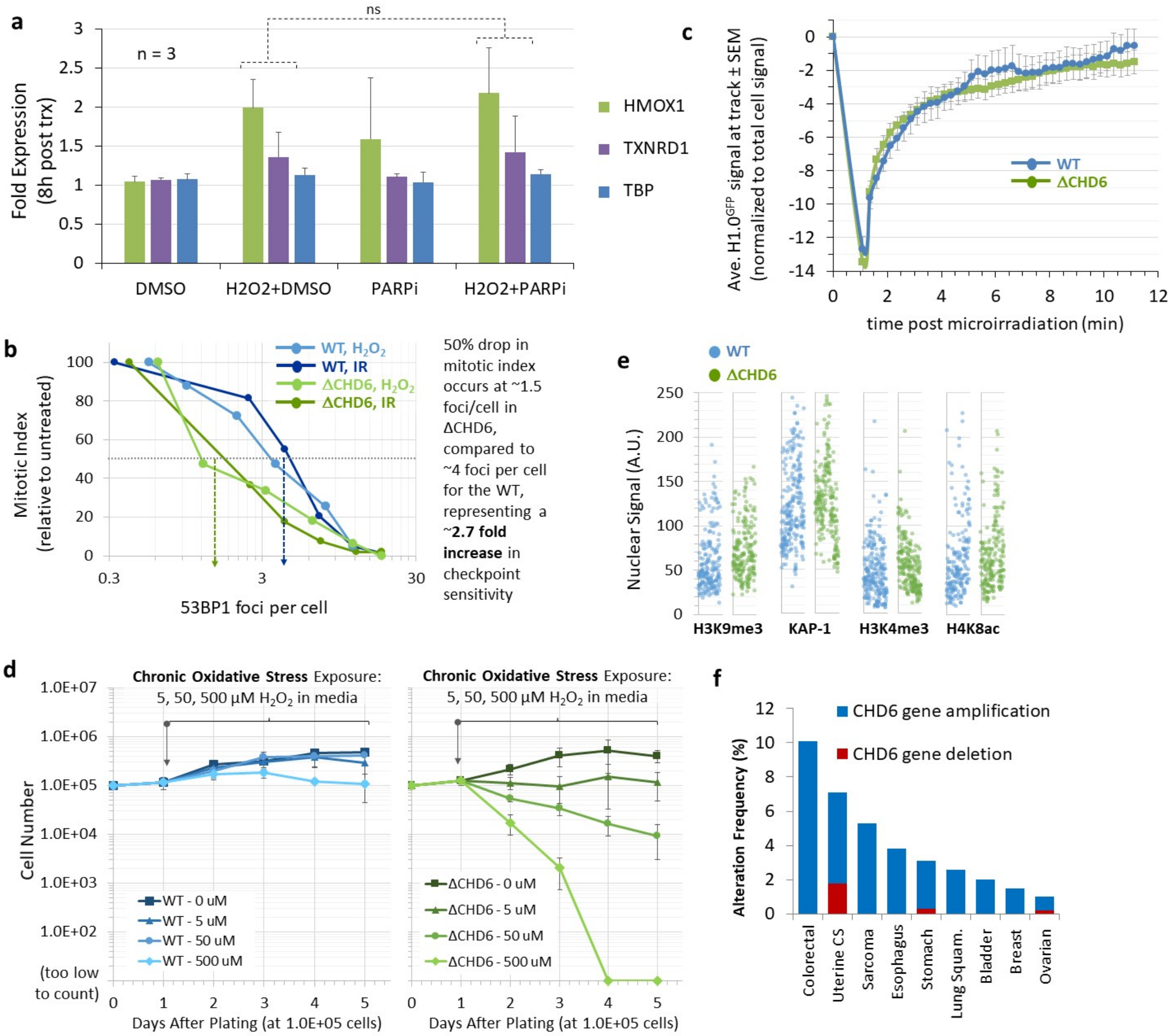

Supplementary Figure 4: Phenotypes of CHD6 ablated cells continued. A: A549 cells were exposed to 0 or 500 µM H_2O_2 in media for 1 day in the presence or absence of PARPi, before extraction and analysis by quantitative PCR to ascertain HMOX1, TXNRD1 and TBP mRNA expression. Errors bars represent s.e.m, n=3, ns = not significant. B: The data in Fig 5A were re-expressed as mitotic index relative to 53BP1 foci number. Arrows denote the foci number at which a 50% reduction in mitotic index is observed. C: A549 cells were transfected with Histone H1.0^{GFP} plasmids and labelled with BrdU; 16 h later, cells were subjected to laser microirradiation and live imaged over 11 min. GFP signal at microirradiation track was quantified as described in the methods. Error bars represent s.e.m, n=10. D: Cells were cultured at 5% O_2. Exactly 1.0E+05 WT (blue lines) and ΔCHD6 (green lines) A549 cells were plated and, 1 day later, 0, 50 or 500 µM H_2O_2 was added to media. Fresh H_2O_2 was added to media every day to maintain chronic exposure. Viable cell numbers were counted daily using a Moxi-Z cell counter. Error bars represent s.e.m, n=3. E: WT (blue) and ΔCHD6 (green) cells were immunostained for the indicated antibodies and quantified by ImageJ, n=3. F: % gene alteration frequency, showing only gene amplification (blue) or deletion (red), for nine human cancers (data derived and adapted from The Cancer Genome Atlas datasets available at cbioportal.org). In all cases, blue represents Parental WT A549s and green represents ΔCHD6 A549s.

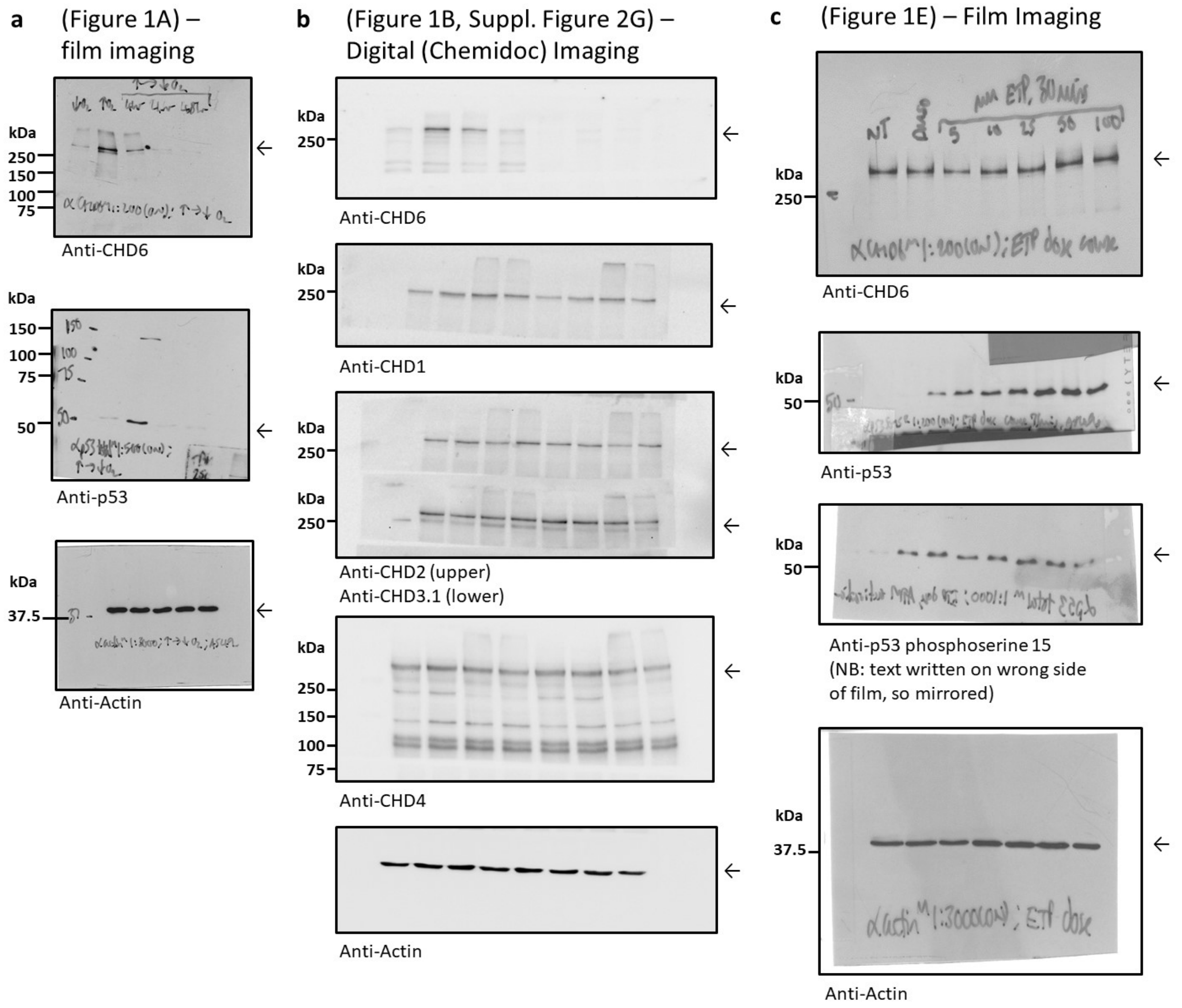

Supplementary Figure 5: Full-size scans of immunoblots. A-C: Uncropped blots for indicated figures. Images based on scans of developed film, versus digital files from ChemiDoc imaging, are indicated.

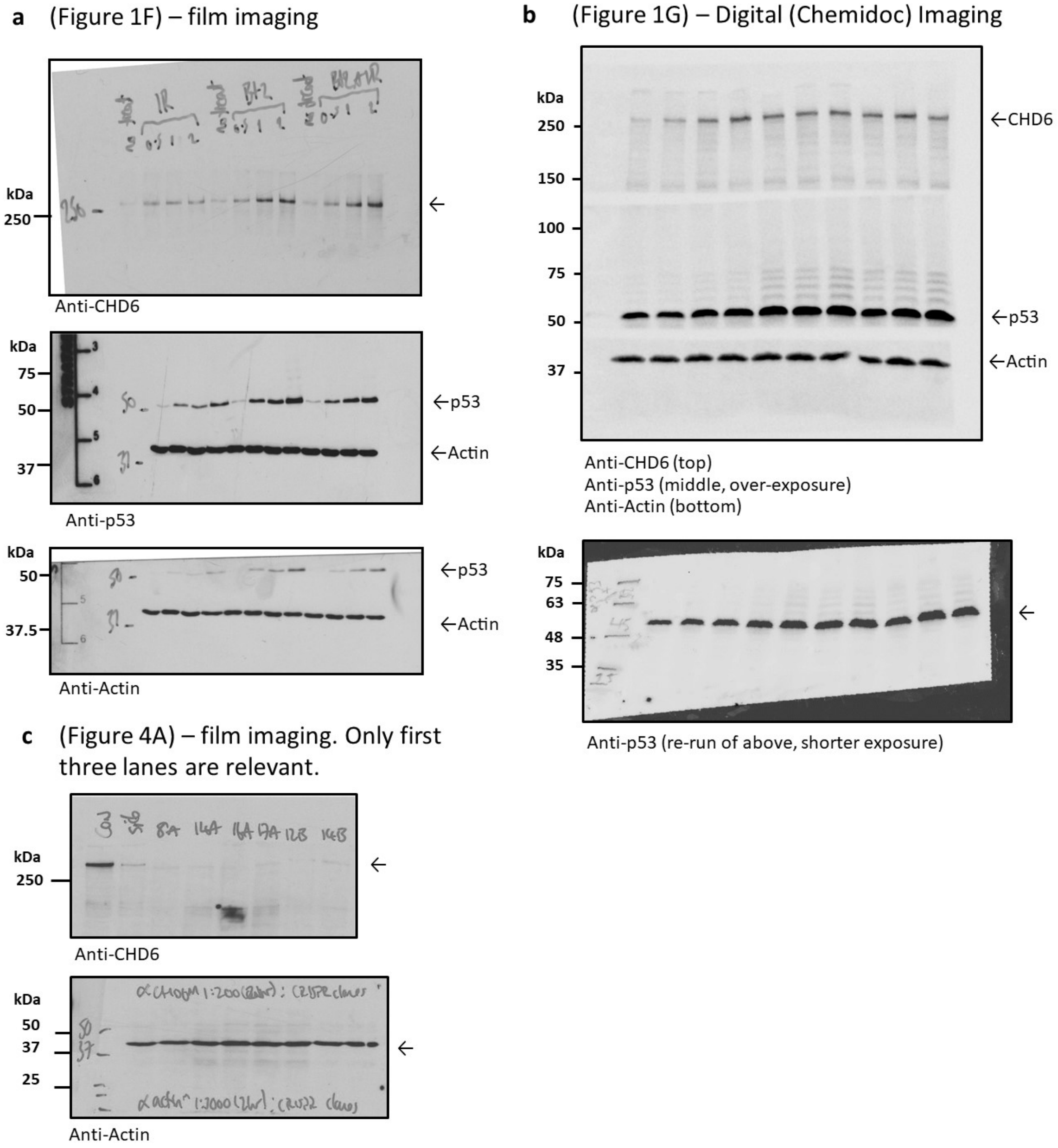

Supplementary Figure 6: Full-size scans of immunoblots. A-C: Uncropped blots for indicated figures. Images based on scans of developed film, versus digital files from ChemiDoc imaging, are indicated.

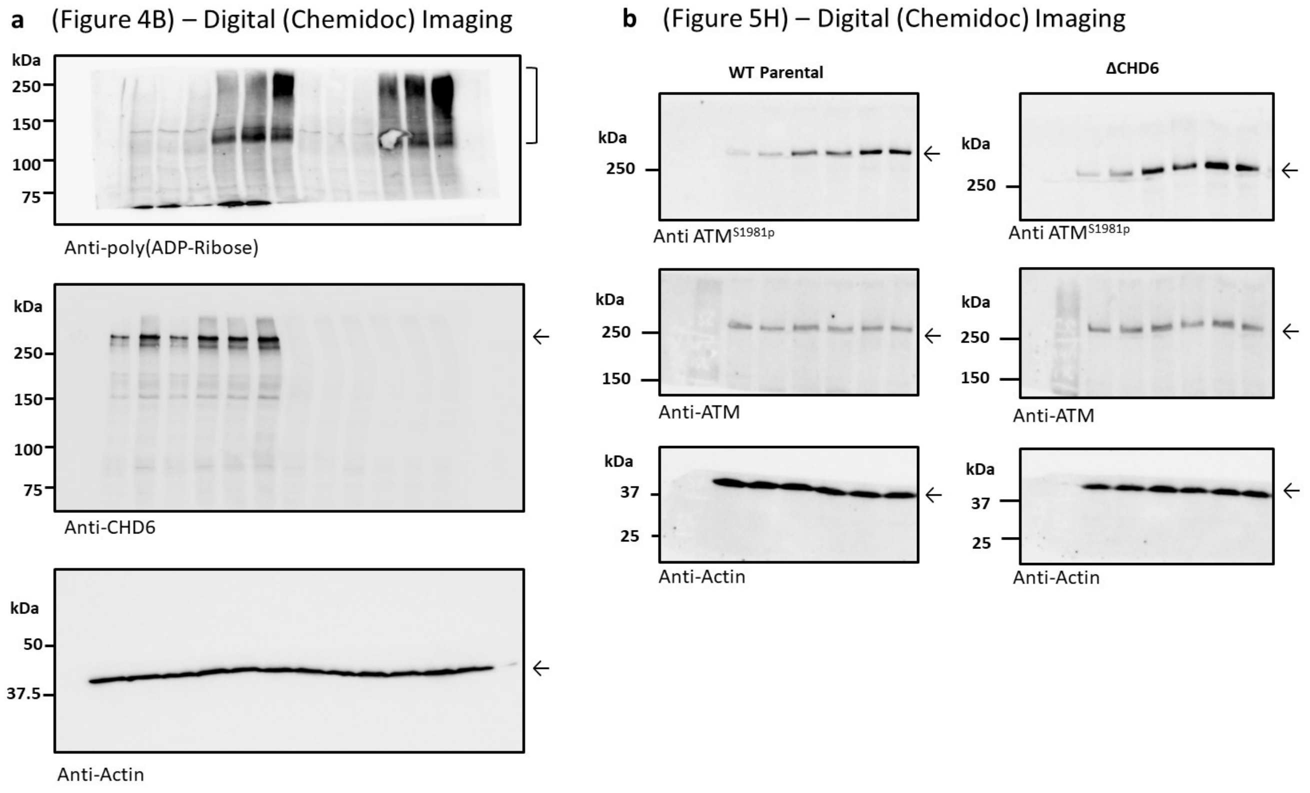

Supplementary Figure 7: Full-size scans of immunoblots. A-B: Uncropped blots for indicated figures. Images based on scans of developed film, versus digital files from ChemiDoc imaging, are indicated.

a (Figure 4E) – FACS sorting protocol and gating

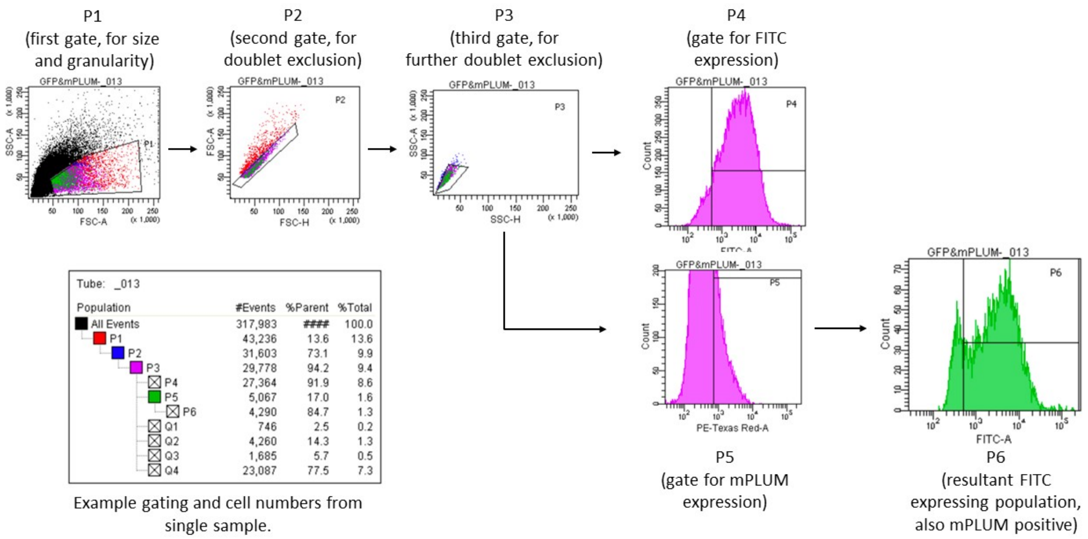

Tube: _013

Population	#Events	%Parent	%Total
All Events	317,983	####	100.0
P1	43,236	13.6	13.6
P2	31,603	73.1	9.9
P3	29,778	94.2	9.4
P4	27,364	91.9	8.6
P5	5,067	17.0	1.6
P6	4,290	84.7	1.3
Q1	746	2.5	0.2
Q2	4,260	14.3	1.3
Q3	1,685	5.7	0.5
Q4	23,087	77.5	7.3

Example gating and cell numbers from single sample.

b (Figure 5G) – FACS sorting protocol and gating

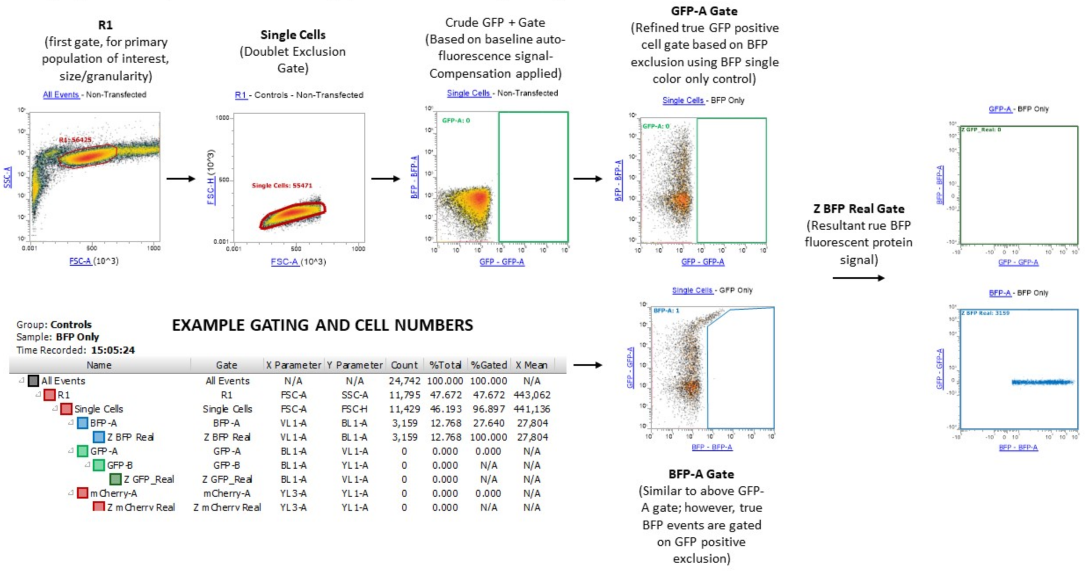

EXAMPLE GATING AND CELL NUMBERS

Group: **Controls**
Sample: **BFP Only**
Time Recorded: **15:05:24**

Name	Gate	X Parameter	Y Parameter	Count	%Total	%Gated	X Mean
All Events	All Events	N/A	N/A	24,742	100.000	100.000	N/A
R1	R1	FSC-A	SSC-A	11,795	47.672	47.672	443,062
Single Cells	Single Cells	FSC-A	FSC-H	11,429	46.193	96.897	441,136
BFP-A	BFP-A	VL1-A	BL1-A	3,159	12.768	27.640	27,804
Z BFP Real	Z BFP Real	VL1-A	BL1-A	3,159	12.768	100.000	27,804
GFP-A	GFP-A	BL1-A	VL1-A	0	0.000	0.000	N/A
GFP-B	GFP-B	BL1-A	YL1-A	0	0.000	N/A	N/A
Z GFP_Real	Z GFP_Real	BL1-A	VL1-A	0	0.000	N/A	N/A
mCherry-A	mCherry-A	YL3-A	YL1-A	0	0.000	0.000	N/A
Z mCherry Real	Z mCherry Real	YL3-A	YL1-A	0	0.000	N/A	N/A

Supplementary Figure 8: Gating and Protocol of FACS analysis. A: Additional FACS sorting methodology for data described in Figure 4e. Panel B: Additional FACS sorting methodology for data described in Figure 5g.

Supplementary Table 1: Statistic analysis of laser microirradiation data. Corresponding to CHD6 truncation mutants in Fig 3e, indicating 2-way ANOVAs comparing entire curves, then specific comparison of data at selected time points across distinct periods within the kinetics of CHD6 recruitment and dispersal (values mean the following: ns = p>0.05 ; *p<0.05; **p<0.01; ***p<0.001; ****p<0.0001). 4 minutes correlates with the end of the early, PAR-dependent recruitment. 8 min correlates with end of the slower period of recruitment, and maximum signal reached. 14 min represents the period of stable retention, whilst 21 min represents the period of significant dispersal.

**Comparison**	**Two-way Anova**	**4 min**	**8 min**	**14 min**	**21 min**
WT v PARPi	****	****	****	****	ns
WT v PARGi	****	ns	*	****	****
siScr v siPARP1+2	****	****	***	ns	ns
siScr v siPARG	****	ns	ns	ns	ns
WT v K492Q	ns	ns	ns	ns	ns
WT v ΔCD1+2	ns	ns	ns	ns	ns
WT v 1-1448	ns	ns	ns	ns	ns
WT v 1-1028	ns	ns	ns	ns	ns
WT v 1-449	****	ns	ns	ns	ns
WT v 1-269	****	ns	***	*	ns
WT v 1-231	****	ns	**	**	ns
WT v 1-171	****	****	****	****	**
PARPi v 1-171	ns	ns	ns	ns	ns

Supplementary Table 2: Primary antibodies used in this study.

Antibody	Host	Company (reference #)	IF	IB
53BP1	Rabbit	Abcam, ab21083	1:800	
GFP	Rabbit	Abcam, ab290		1:500
CHD6	Mouse	Abcam, ab51330		1:200
CHD3	Rabbit	Abcam, ab84528		1:500
CHD4	Mouse	Abcam, ab54603		1:400
p53	Mouse	Calbiochem, OP43		1:800
Actin	Mouse	Abcam, ab3280		1:300
ATMS1981p	Rabbit	Abcam, ab 81292		1:2000
H3S10p	Mouse	Abcam, ab 14955	1:200	
XRCC1	Rabbit	Abcam, ab47920	1:2000	
PAR	Rabbit	Trevigen, 4336-PBC-100	1:400	1:1000
CHD1	Mouse	Santa Cruz, sc-271626		1:500
PARP1	Rabbit	ENZO, ALX-210-302		1:1000
PARP2	Mouse	ENZO, ALX-804-639		1:500
XRCC4	Rabbit	Gift from Dr. Modesti	1:500	
γH2AX	Mouse	Millipore, clone JBW301	1:2000	
CHD2	Rat	Millipore, MAB E873		1:500
ATM	Rabbit	Clone ATM4BA (Klement et al 2014)		1:2000
CHD6	Mouse	LSBio, LSC342418	1:50	
KAP-1	Rabbit	Abcam, ab10584	1:800	
γH2AX	Mouse	Abcam, ab26350	1:800	

Supplementary Table 3: siRNA sequences.

siRNA	Sequence (5'-3')	coding region targetted (nt)
siScrambled	CGGCAUCAAGAGUACGCAAAGAGUA	na
siCHD6 A	GGACAGAGAUGAAUGCCAUUGUGUA	1531-1556
siCHD6 B	CAGACCGCUUUGUCUUUCUUCUGUG	2548-2573
siPARP1 A	AAGAAAGUGUGUUCAACUAAUGACC	928-952
siPARP1 B	UUAAGAUAGAGCGUGAAGGCGAAUG	2681-2706
siPARP2	CGAAGGAUUGCUUCAAGGUAAUUAC	1543-1567
siPARG A	UUACCAGUUGGAUGGACACUAAAGG	316-340
siPARG B	GAAGAUGGUAGUUCCUCCCAAACAG	1067-1092
siXRCC1 A	GGAGACCAUCUCUGUGGUCCUACAG	240-265
siXRCC1 B	AACCCGGUCACUCAUAUAGUCCUCG	1839-1864
siCHD3 A	GGGCCAUCAUUCGUGAGAAUGAAUU	2618-2643
siCHD3 B	AGGCACAGGUGAAGUUCCAUGUUCU	2699-2724

Supplementary Table 4: GFP-tagged CHD6 plasmid mutations.

GFP mutant	Amino Acid position(s)	Nucleotide position(s)	Codon change
K492Q	K492>Q	1471-1475	AAA>CAA
ΔCD1+2	F318>A, Y322>A, Y398>A, W402>A	352-354, 364-366, 1191-1193, 1204-1206	TTC>GCC, TAT>GCT, TAC>GCC, TGG>GCG
1-1448		4044-4046	TGT>TAG
1-1028		3085-3087	ATA>TAG
1-449		1347-1349	GAG>TGA
1-269		807-809	CGA>TAG
1-231		693-695	GAC>TAG
1-171		513-515	TCG>TAG
1-108		355-357	AAG>TAG

Supplementary Table 5: Primer sequences for qPCR analysis.

Target	Forward primer 5'-3'	Reverse primer 5'-3'
NQO1	TGAAGAAGAAAGGATGGGAGGT	GGCCTTCTTTATAAGCCAGAACA
HMOX1	TGCTGACCCATGACACCAAG	GGGCAGAATCTTGCACTTTGTT
TXRND1	CCAGGCCGACTCAGAGTAG	GCCAGCATCACCGTATTATATTCTC
G6PD	CCCGGAAACGGTCGTACACT	CATGACGCTGTCTGCGCTTC
GSTM2	CCTTCCCAAACCTGAAGGA	TTCAAGGCCCTACTTGTTGC
GPX2	TTTCAATACGTTCCGGGGCA	TCTGACAGTTCTCCTGATGTCC
GAPDH	GAAGGTCGGAGTCAACGGATTT	ATGGGTGGAATCATATTGGAAC
TBP	CGCCAGCTTCGGAGAGTTC	ACAACCAAGATTCACTGTGGATACA

Supplementary Table 6: DNA lesions used in multiplexed DNA repair assay.

Repair Pathway (lesion)	Lesion-Containing Reporter (REF)	Undamaged Control
NHEJ (ScaI-induced DSB)	BFP_NHEJ [1]	pmax_BFP
HR (StuI-Induced DSB)	D5GFP_StuI_Linear [1,2]	pCX-NNX-GFP
NER (800 J/m^2 UVC-Induced Lesions)	mOrange_UV [1]	pmax_mOrange
MMR (G:G mismatch)	mOrange_GG [1]	pmax_mOrange
BER (Tetrahydrofuran)	GFP_THF [3]	pmax_GFP

SUPPLEMENTARY REFERENCES

1. Nagel, ZD et al. Multiplexed DNA repair assays for multiple lesions and multiple doses via transcription inhibition and transcriptional mutagenesis. *Proc Natl Acad Sci U S A* **111**, E1823-32 (2014).
2. Kiziltepe, T et al. Delineation of the chemical pathways underlying nitric oxide-induced homologous recombination in mammalian cells. *Chemistry & Biology* **12**, 357-369 (2005).
3. Chaim, IA et al. In vivo measurements of interindividual differences in DNA glycosylases and APE1 activities. *Proc Natl Acad Sci U S A* **114**, E10379-E10388 (2017).
