# Supplementary material for: The CHD6 chromatin remodeler is an oxidative DNA damage response factor
Source: Nat Commun. 2019 Jan 16;10:241. doi: 10.1038/s41467-018-08111-y (PMC6335469; doi:10.1038/s41467-018-08111-y)
Supplement: Supplementary file 3 — Reporting Summary [file 41467_2018_8111_MOESM3_ESM.pdf]

nature research

Corresponding author(s): Aaron A. Goodarzi

Reporting Summary

Nature Research wishes to improve the reproducibility of the work that we publish. This form provides structure for consistency and transparency in reporting. For further information on Nature Research policies, see Authors & Referees and the Editorial Policy Checklist.

Statistical parameters

When statistical analyses are reported, confirm that the following items are present in the relevant location (e.g. figure legend, table legend, main text, or Methods section).

n/a	Confirmed	
☐	☒	The exact sample size (n) for each experimental group/condition, given as a discrete number and unit of measurement
☐	☒	An indication of whether measurements were taken from distinct samples or whether the same sample was measured repeatedly
☐	☒	The statistical test(s) used AND whether they are one- or two-sided<br>*Only common tests should be described solely by name; describe more complex techniques in the Methods section.*
☒	☐	A description of all covariates tested
☒	☐	A description of any assumptions or corrections, such as tests of normality and adjustment for multiple comparisons
☐	☒	A full description of the statistics including central tendency (e.g. means) or other basic estimates (e.g. regression coefficient) AND variation (e.g. standard deviation) or associated estimates of uncertainty (e.g. confidence intervals)
☐	☒	For null hypothesis testing, the test statistic (e.g. F, t, r) with confidence intervals, effect sizes, degrees of freedom and P value noted<br>*Give P values as exact values whenever suitable.*
☒	☐	For Bayesian analysis, information on the choice of priors and Markov chain Monte Carlo settings
☒	☐	For hierarchical and complex designs, identification of the appropriate level for tests and full reporting of outcomes
☒	☐	Estimates of effect sizes (e.g. Cohen's d, Pearson's r), indicating how they were calculated
☐	☒	Clearly defined error bars<br>*State explicitly what error bars represent (e.g. SD, SE, CI)*

Our web collection on statistics for biologists may be useful.

Software and code

Policy information about availability of computer code

Data collection	No software used
Data analysis	No software used

For manuscripts utilizing custom algorithms or software that are central to the research but not yet described in published literature, software must be made available to editors/reviewers upon request. We strongly encourage code deposition in a community repository (e.g. GitHub). See the Nature Research guidelines for submitting code & software for further information.

Data

Policy information about availability of data

All manuscripts must include a data availability statement. This statement should provide the following information, where applicable:
- Accession codes, unique identifiers, or web links for publicly available datasets
- A list of figures that have associated raw data
- A description of any restrictions on data availability

The data sets generated and/or analyzed during the current study are available from the corresponding author on reasonable request.

Field-specific reporting

Please select the best fit for your research. If you are not sure, read the appropriate sections before making your selection.

☒ Life sciences ☐ Behavioural & social sciences ☐ Ecological, evolutionary & environmental sciences

For a reference copy of the document with all sections, see nature.com/authors/policies/ReportingSummary-flat.pdf

Life sciences study design

All studies must disclose on these points even when the disclosure is negative.

Sample size	At least three independent replicates were collected for robust statistical analysis.
Data exclusions	No data was excluded at the analysis stage.
Replication	Experiments were repeated independently three or more times and small experimental variance was confirmed. All data was reproducible.
Randomization	N/A
Blinding	Certain experiments prone to conformation bias were blinded, for example the comet assay.

Reporting for specific materials, systems and methods

Materials & experimental systems

n/a	Involved in the study	
☐	☒	Unique biological materials
☐	☒	Antibodies
☐	☒	Eukaryotic cell lines
☒	☐	Palaeontology
☒	☐	Animals and other organisms
☒	☐	Human research participants

Methods

n/a	Involved in the study	
☒	☐	ChIP-seq
☐	☒	Flow cytometry
☒	☐	MRI-based neuroimaging

Unique biological materials

Policy information about availability of materials

Obtaining unique materials	The FM-HCR plasmids generated and/or analyzed during the current study may be available from the corresponding author on reasonable request and availability.

Antibodies

Antibodies used	Supplementary table 2
Validation	Supplementary table 2

Eukaryotic cell lines

Policy information about cell lines

Cell line source(s)	ATCC
Authentication	A549 cells were sequenced and matched the reference genome. CRISPR generated cell lines were sequenced to determine genetic alteration within the CHD6 genomic region.
Mycoplasma contamination	Cell lines tested every 3 months for mycoplasma contamination. All cell lines negative for mycoplasma contamination using the SIGMA test kit.

Commonly misidentified lines
(See ICLAC register)

N/A

Flow Cytometry

Plots

Confirm that:

☐ The axis labels state the marker and fluorochrome used (e.g. CD4-FITC).

☐ The axis scales are clearly visible. Include numbers along axes only for bottom left plot of group (a 'group' is an analysis of identical markers).

☐ All plots are contour plots with outliers or pseudocolor plots.

☐ A numerical value for number of cells or percentage (with statistics) is provided.

Methodology

Sample preparation	Oxidative stress asssay: Briefly, H2DCFDA dye (20 uM) was loaded into cells at 37 degrees celsius in phenol red-free and serum-free DMEM for 1 hour. Cells were rinsed with PBS three times, returned to their media, and treated with peroxide. Cells were then rinsed and collected in cold PBS, and any extracellular dye was quenched with 0.0025% Trypan blue (v/v%). Cells were then resuspended in 250 uL of cold PBS, and submitted for FACS analysis.<br>DNA repair assay, FM-HCR: Briefly, cells were collected via trypsinization (counted) and pelleted via centrifugation. Media was aspirated, and pellets were resuspended in PBS (Ca/Mg free), pelleted via centrifugation, and PBS was aspirated. Cells were resuspended in Neon electroporation buffer (Buffer R), and cells were then transfected with FM-HCR plasmid cocktails using a Neon Electroporator system using manufacturer recommended settings for A549 cells. Cells were incubated at 37 degrees celsius in DMEM for 24 hours. At which time, cells were collected via trypsinization (200ul) and neutralized with 320ul DMEM cell media. Samples (500ul) were then run through the Attune NxT flow cytometer.
Instrument	Oxidative stress assay: BD Bioscience LSRII Flow Cytometer. FM-HCR: Attune NxT Flow Cytometer
Software	Oxidative stress assay: FACSDiva version 6.1.3. FM-HCR: Attune NxT Software (version 2.7.873.0)
Cell population abundance	For the H2DCFDA Oxidative Stress examinations: after the first three gating events to eliminate cellular debris and doublets, the post-sort fractions are approximately 8-15% of the total number of events (all cells, debris, doublets, etc, P1-P3). This fraction went on for further analysis and gating. The area scaling was set with cells from our experiments for the best possible analysis.<br>For FM-HCR, the main population of live cells represented an isogenic cell population (of either A549 or delta CHD6 cell lines). The main population scatter was reduced to exclude doublet cells prior to further sub-gating stringencies based on fluorescent protein expression levels.
Gating strategy	For the H2DCFDA Oxidative Stress examinations: several sequential gating steps were used in these experiments to examine only the cell populations of interest. The first three gates applied were to exclude cellular debris and doublets as per standard side and forward scatter gating procedures, via area scaling (P1, P2 and P3). Further gates were then applied to examine the proportions of H2DCFDA (FITC) positive cells and the mean fluorescence intensities, with the thresholds for these gates set with unstained cell samples (to account for autofluorescence) (P4). In the case of CHD6-mPLUM transfected cells, a further gate was applied (P5) that identified cells expressing mPLUM, to examine the DCFDA fluorescence only in mPLUM-positive cell populations (P6).<br>For FM-HCR, the main cell population body was gated with denotation "R1", which was then used to nest a "Single Cells" gate to exclude cell doublets. Single color controls for all fluorescent proteins (FP), such as BFP, GFP, mOrange, mPlum, mCherry were transfected and run via flow when applicable in reference to an un-transfected control. Autofluorescence background signal, and FP signals were corrected in the following steps. Attune NxT Software was used to manually set compensation values for each single fluorescent protein in reference to each of the other FP's/filters in a matrix-wise manner. Channel "leaking" for the FP's was adjusted accordingly (i.e. GFP-A), and sub-gates were used as appropriate (i.e. GFP-B, etc) to ensure less than 0.1% channel leaking from any given FP in single color controls. Note: Single color controls were transfected at a higher concentration than any given damaged FM-HCR plasmid (Ex. 500ng of each single color control was transfected to determine compensation, while the most concentrated damaged FM-HCR plasmid was 100ng), therefore we are confident in the stringency of our gating scheme to discriminate false positives and/or false negatives. Lastly, true positive events were denoted as "Z True FP" as an endpoint quantified gate for each respective FP color. FM-HCR assays were quantified according to the methods section.

☒ Tick this box to confirm that a figure exemplifying the gating strategy is provided in the Supplementary Information.
